# High Anti-*Leishmania* IgG Antibody Levels Are Associated With Severity of Mucosal Leishmaniasis

**DOI:** 10.3389/fcimb.2021.652956

**Published:** 2021-04-09

**Authors:** Clara Mônica F. de Lima, Andrea S. Magalhães, Rúbia Costa, Carolina C. Barreto, Paulo R. L. Machado, Edgar M. Carvalho, Marcus M. Lessa, Lucas P. Carvalho

**Affiliations:** ^1^ Serviço de Imunologia, Hospital Universitário Prof. Edgard Santos, Universidade Federal da Bahia, Salvador, Brazil; ^2^ School of Medicine, Programa de Pós-graduação em Ciências da Saúde - Universidade Federal da Bahia, Salvador, Brazil; ^3^ Ministry of Sciences and Technology, Instituto Nacional de Ciência e Tecnologia em Doenças Tropicais, INCT-DT, Salvador, Brazil; ^4^ Laboratório de Pesquisas Clínicas (LAPEC), Instituto Gonçalo Moniz (IGM), Fiocruz, Salvador, Brazil

**Keywords:** mucosal leishmaniasis, antibodies, IgG subclasses, *Leishmania braziliensis*, therapeutic failure

## Abstract

**Background:**

Mucosal leishmaniasis (ML), the most inflammatory form of tegumentary leishmaniasis, is predominantly caused by *Leishmania braziliensis*. The disease is characterized by the development of lesions, mainly in the nasal mucosa. An exacerbated inflammatory response has been associated with the presence of destructive and disfiguring lesions, with stages of severity ranging from small nodulations to the complete destruction of the nasal pyramid architecture. As *Leishmania* is an intracellular parasite, most immunological studies have emphasized the cell-mediated immune response, while relatively few studies aimed to investigate the role antibodies in protection against, or the pathology of ML.

**Methods:**

Patients with a confirmed diagnosis of ML were classified according to clinical staging criteria. Serum levels of *Leishmania*-specific IgG, IgG1 and IgG2 antibodies were determined by ELISA before and after treatment with antimony or antimony plus pentoxifylline.

**Results:**

Patients in stages IV and V produced higher concentrations of IgG and IgG1 antibodies when compared to those in stage I and II. Significant reductions were seen in the concentrations of IgG and IgG2 antibodies in most patients who responded well to treatment.

**Conclusions:**

Our data demonstrate an association between IgG antibody titers and the severity of mucosal disease. The observed reduction in antibody production after successful treatment in most patients preliminarily indicates that these tests can be used to aid in the assessment of therapeutic response.

## Introduction

American tegumentary leishmaniasis (ATL) is widely distributed around the world, and often presents high morbidity due to the possibility of developing destructive lesions that can disfigure and disable individuals, significantly impacting their quality of life (World Health Organization - Control of Leishmaniasis, 2010). In endemic areas of *Leishmania braziliensis* transmission, mucosal leishmaniasis (ML), a disease also known as mucocutaneous leishmaniasis, occurs in 3% of patients concomitantly or following the cure of cutaneous leishmaniasis (CL). ML can also be caused by other *Leishmania* species, such as *Leishmania amazonensis* and *Leishmania guaianensis* (Grimaldi et al., 1987). In about 15% of cases, no previous history or scarring due to CL is documented (Boaventura et al., 2006; Miranda Lessa et al., 2007). Moreover, up to 40% of patients with an emergent disease termed disseminated cutaneous leishmaniasis (DL) have mucosal involvement (Carvalho et al., 1994). While ML primarily affects the nasal mucosa (90% of cases), the second most affected site is the pharyngeal mucosa, followed by the laryngeal mucosal and oral cavity (Marsden, 1994; Miranda Lessa et al., 2007). Importantly, the involvement of these latter areas is an indicator of disease severity (Marsden, 1994; Miranda Lessa et al., 2007). Our group proposed staging criteria (I to V) for patients who only have nasal mucosal involvement. Stage I is characterized by nodulation in the absence of ulceration in the mucosa of the nasal septum; stage II: superficial ulceration; stage III: deep ulceration; stage IV: nasal septum perforation; stage V: destruction of the nasal pyramid architecture (Lessa et al., 2012).

As *Leishmania* is an intracellular parasite, the role of the innate and T cell responses in the pathogenesis of disease has been widely studied. Lymphocytes from ML patients were found to be more proliferative than those from CL patients when stimulated with *Leishmania* antigens (Carvalho et al., 2007), and the production of cytokines/chemokines by macrophages and T cells, such as TNF, IFN-γ and CXCL9, was observed to be higher in ML compared to CL (Bacellar et al., 2002; Junqueira Pedras et al., 2003). The impairment in regulatory mechanisms that may attenuate the immune response also play an important role in the exaggerated inflammatory response observed in ML, as Cells from ML patients produce less IL-10 and its receptor than those from CL. The role of antibodies in *Leishmania* has not been completely elucidated. High levels of antibodies are produced in all clinical forms of leishmaniasis, and high levels of IgG and IgE antibodies are found in ML (Junqueira Pedras et al., 2003).

In the present study, we observed that antibodies against *Leishmania* antigens were associated with ML severity, and the data suggest that therapeutic success may be associated with decreases in antibody levels.

## Materials and Methods

### Study Design

A longitudinal study was performed to investigate the association between IgG titers and disease severity, as well as response to therapy in ML patients. Clinical stages were determined upon ENT examination and classified from stages I to V according to the criteria established by Lessa et al. (2012). The production of anti-*Leishmania* antigens antibodies was determined before and after therapy. Treatment was considered successful upon the complete re-epithelization of nasal cavity lesions (clinical cure).

### Study Area and Patients

This study received approval (CAAE: 01229212.0.0000.0049) from the Institutional Review Board of the Professor Edgard Santos University Hospital (HUPES-UFBA), Salvador, Bahia-Brazil. This study was carried in an area endemic for CL, Corte de Pedra, located in southeastern Bahia, Brazil. This region is home to a municipal clinic that houses a reference center for tegumentary leishmaniasis diagnosis and treatment. Diagnosis was based on the presence of nodules and ulcers in the nasal mucosa, and by the presence of *Leishmania* DNA as confirmed by PCR (Weirather et al., 2011). Twenty-seven ML patients were enrolled in this study, six of whom received pentavalent antimony alone (20 mg/Kg/day for 30 days), while 21 patients were treated with a combined regimen consisting of pentavalent antimony with pentoxifylline (1200 mg/day orally for 30 days). To evaluate serological test specificity using a soluble antigen of *Leishmania brazilliensis* (SLA) in patients with ML, serum from 25 patients with cutaneous leishmaniasis (CL) were used as controls. Clinical and serological evaluations were performed in ML patients prior to and 90 days following the onset of therapy.

### Soluble *Leishmania* Antigen

A *L. braziliensis* strain (MHOM/BR/2001), isolated from a patient with ML, was used to prepare soluble *Leishmania* antigen by sonication and centrifugation, as previously described (Reed et al., 1986). SLA was used at a concentration of 5μg/mL, after testing negative for endotoxins using the Limulus amebocyte lysate test.

### Detection of Anti-Leishmanial IgG Antibodies by ELISA

Peripheral blood from ML patients was added to a dry tube for serum separation and stored at -20°C. Polystyrene plates were sensitized with 5 μg/well of soluble *Leishmania* antigen in carbonate/bicarbonate buffer and incubated overnight at 4°C. To block unspecific antibody binding, 1% bovine albumin (BSA) in PBS was added for 1 hour at 37°C. Sera (1:50 dilution) from patients and healthy controls were added and incubated for 1 hour at 37°C. Peroxidase-conjugated monoclonal anti-human IgG (Sigma-Aldrich, St. Louis, MO, USA), or monoclonal anti-human IgG1 and IgG2 (Sigma-Aldrich, St. Louis, MO, USA) were then added. After each step described above, 4-7 washes were performed with phosphate buffered saline containing 0.05% Tween 20. Finally, the enzyme substrate Tetramethylbenzidine (TMB) was added, and after 15 minutes the reaction was quenched with 2N H_2_SO_4_. Readings were performed on a microplate reader at a wavelength of 450 nm, and results were expressed as optical density (OD). The cut off values (total IgG is 0.395; IgG1 0.042; IgG2 0.159) were established by taking into account the mean + 3 standard deviations of absorbance readings from healthy subjects.

### Detection of Cytokines in Serum

Serum was obtained from peripheral blood from 15 ML patients. Determination of IFN-γ and TNF concentrations were performed by ELISA (BD biosciences) according to the manufactures instructions. The results are expressed in pg/ml.

### Statistical Analysis

Non-parametric testing involved the Kruskal-Wallis and Wilcoxon signed-rank tests, for comparisons of continuous variables using GraphPad Prism version 5.0 (GraphPad Software, San Diego, CA, USA). Statistical differences were considered when p values were less than 0.05.

## Results

Twenty-seven patients with ML and 25 with CL were enrolled in this study. Due to the small number of patients classified as stage I or V, patients were divided into three groups: Stages I and II (40.7%), stage III (25.9%) and stages IV and V (33.4%). Regarding sex, males were predominant in most groups, although no statistical significant difference was found between groups. We also found no difference in the frequency of individuals that reached cure within 90 days after therapy initiation, between groups. Ninety-six percent of patients with ML had previous CL lesion whereas no CL patients had history of leishmaniasis.

**Figure 1:**
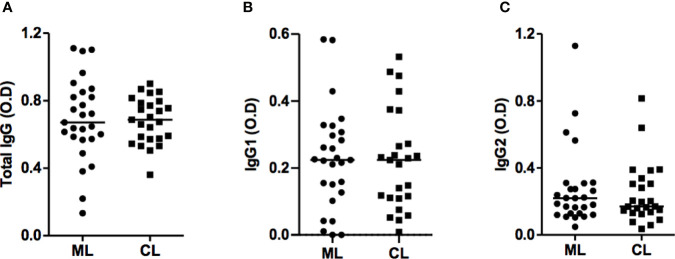
ML and CL patients produce IgG antibodies against *Leishmania braziliensis* antigens. Anti-SLA total IgG **(A)**, IgG1 **(B)** and IgG2 **(C)** titers from ML (n=27) and CL (n=25) patients, performed by ELISA technique. Statistical analysis was performed by the Mann-Whitney U test. Results are expressed in optical density (OD).

**Figure 2:**
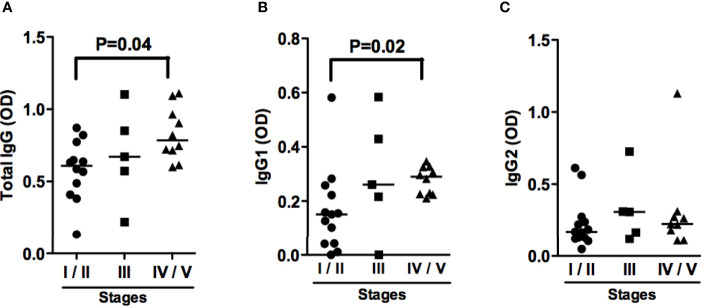
ML patients with severe disease present increased Total IgG and IgG1 antibodies titers. Anti-SLA total IgG **(A)**, IgG1 **(B)** and IgG2 **(C)** titers from ML patients in different stage of the disease, performed by ELISA technique. Statistical analysis was performed by the Kruskal-Wallis test. Results are expressed in optical density (OD). ML stage I/II (n=12); ML stage III (n=5); ML stage IV/V (n=10).

Titers of IgG antibodies are known to increase in active CL and visceral leishmaniasis, and decrease after cure (Fagundes-Silva et al., 2012). Since ML is the most inflammatory form of tegumentary leishmaniasis, we first wanted to investigate whether there were differences among IgG titers between ML and CL patients and found no differences in total IgG, IgG1 and IgG2 titers between these groups (**Figure 1**). We also examined antibodies levels among ML patients in different stages of disease. Interestingly, patients on stages IV and V presented high titers of total IgG and IgG2 when compared to those in stages I and II (**Figure 2**).

**Figure 3:**
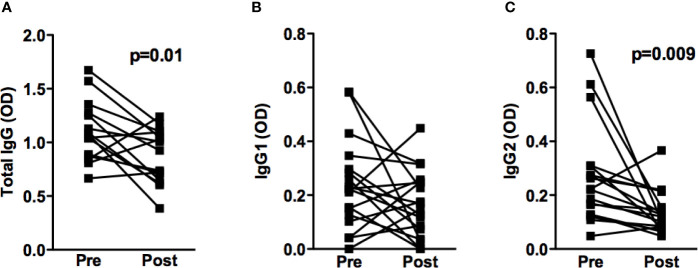
IgG and IgG subclasses titers before and after treatment of ML patients. Anti-SLA total IgG (n=15) **(A)**, IgG1 (n=18) **(B)** and IgG2 (n=18) **(C)** titers from ML patients, performed by ELISA technique. Statistical analysis was performed by the Wilcoxon signed-rank test. Results are expressed in optical density (OD).

To determine whether reduced antibody production was associated with a successful therapeutic response, the titers of *Leishmania*-specific IgG, IgG1 and IgG2 were determined before and 90 days following the onset of therapy in 18 ML patients. Reductions were observed in the absorbance of total IgG, in 73% of patients, and IgG2, in 89% of patients who successfully responded to antimonial treatment (**Figure 3**). Interestingly, increase in the levels of IgG, IgG1 and IgG2 was observed in the three individuals that failed therapy.

**Figure 4:**
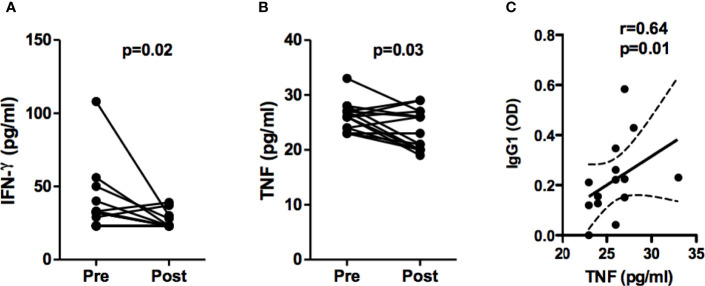
Serum levels of IFN-γ and TNF from ML patients. Serum concentration of IFN-γ **(A)** and TNF **(B)** from ML patients (n=15) were assessed before and 90 days after pentavalent antimony therapy, by ELISA technique. Statistical analysis was performed by the Wilcoxon signed-rank test. Results are expressed in pg/ml. **(C)** Spearman correlation between TNF and IgG1 serum concentration, before treatment.

We have previously documented that mononuclear cells from ML patients produce high levels of IFN-γ and inflammatory cytokines in response to *Leishmania* antigens (Bacellar et al., 2002). It is known that IFN-γ induces IgG2 production and IgG induces TNF in monocytes through Fcγ III (Kawano and Noma, 1996; Chouchakova et al., 2001; Chakraborty et al., 2021). Here we found that serum levels of IFN-γ and TNF in ML patients are increased during disease and decreases after treatment, and a positive correlation between pre-treatment TNF and pre-treatment IgG1 serum levels was observed (**Figure 4**).

## Discussion

Although ML, which predominantly affects the nasal mucosa, only occurs in less than 5% of individuals with American tegumentary leishmaniasis, its importance is recognized by the potential to develop destructive nasal lesions, which could then spread to the face. Also, high rates of treatment failure have been documented, not only using pentavalent antimony, but also in response to other leishmanicidal drugs, such as amphotericin B and miltefosine (Soto et al., 2005; Machado et al., 2010). We previously showed that the combination of pentoxifylline with antimony increases ML cure rates and cures patients refractory to antimony. The high rate of cure observed herein stands in agreement with a previous study in which pentavalent antimony in association with pentoxifylline resulted in 100% of included ML patients evolving to cure (Machado et al., 2007).

Most immunological studies in patients with tegumentary leishmaniasis place emphasis on the cell-mediated response, while few investigations have focused on the role of immunoglobulins in protection against or severity of disease. The majority of studies involving the latter area, are concerned with the use of antibodies in serological testing leishmaniasis diagnostic purposes. To investigate associations between the severity of mucosal disease and elevated levels of antibodies against soluble *Leishmania braziliensis* antigen, we compared titers of IgG, IgG1 and IgG2 in the sera of patients with CL and ML prior to and following the onset of treatment, but found no differences between these clinical forms.

Interestingly, we documented an association between high levels of anti-leishmania IgG and IgG1 subclass antibodies with more advanced stages of ML, which suggests either the participation of antibodies in disease severity, or that the antibody absorbance represents a marker of severity. Antibodies may participate in the pathology of leishmaniasis by way of two mechanisms: 1- Opsonization, which increases parasite uptake by macrophages and, consequently, increases parasite burden within these cells; 2- Through increased antibody-mediated cytotoxicity, i.e. higher rates of infected macrophage killing lead to the release of *Leishmania* antigens (Barral-Netto et al., 1995). Both mechanisms can result in increases in inflammatory response and tissue damage. IFN-γ is known to induce IgG2 production, and IgG induces TNF through Fcγ III (CD16). We previously documented increase in the frequency of CD16+ circulating monocytes in CL patients and found that CD16+ monocytes are the main source of TNF and IL-1β in CL patients (Passos et al., 2015; Santos et al., 2018). In the present work we found a positive correlation between systemic concentrations of TNF and IgG1 antibodies, suggesting a contribution of IgG1 to the deleterious inflammatory response and immunopathology observed in ML.

The lack of markers of response to treatment in cutaneous leishmaniasis continues to present challenges to successful therapeutic intervention. Here we documented a drop in the levels of IgG and IgG2 in most ML patients between 60 and 90 days after treatment initiation. Reduced IgG antibody production after cure has also been observed in patients with CL (Schubach et al., 1998; Mendonca et al., 2004). To further investigate the potential applicability of antibodies as markers of therapeutic response in ML, new studies must be conducted involving higher numbers of patients with more extensive timepoint antibody measurements.

One limitation of the present study was the small number of patients evaluated after therapy. However, we were able to clearly demonstrate higher levels of IgG and IgG1 anti-*Leishmania* antibodies in more advanced stages of mucosal nasal disease, in which patients have already developed deep ulcers or septal perforation. Moreover, as the levels of these immunoglobulins drop in most patients after the onset of therapy, antibody measurements may serve as a biomarker of ML prognosis, as well as be a tool capable of supporting the decision to interrupt follow-up in outpatient settings.

Although current serological diagnostic capabilities cannot differentiate between patients with ML and those with CL, the presently established association between antibody absorbance and clinical disease staging suggests that antibodies do indeed participate in the pathogenesis of ML. In addition, the observed reductions in antibody production after treatment and cure provides a preliminary indication that these tests may prove useful in assessing therapeutic response.

## Data Availability Statement

The datasets presented in this study can be found in online repositories. The names of the repository/repositories and accession number(s) can be found below: https://doi.org/10.6084/m9.figshare.13415183.v1.

## Ethics Statement

The studies involving human participants were reviewed and approved by Professor Edgard Santos University Hospital (HUPES-UFBA), Salvador, Bahia-Brazil. The patients/participants provided their written informed consent to participate in this study.

## Author Contributions

CL, LC, ML, RC, CC, and EC conceived and designed the study. CL, LC, AM, and RC analyzed the data. CL, LC, AM, and EC interpreted the data. CL, LC, AM, RC, and CC drafted the manuscript. CL, LC, ML, AM, RC, and EC critically revised the manuscript for intellectual content. EC is guarantor of the manuscript. All authors contributed to the article and approved the submitted version.

## Funding

This study was supported by the US National Institutes of Health grant AI-136032.

## Conflict of Interest

The authors declare that the research was conducted in the absence of any commercial or financial relationships that could be construed as a potential conflict of interest.
